# Trophic Resource Use by Sympatric vs. Allopatric Pelomedusid Turtles in West African Forest Waterbodies

**DOI:** 10.3390/biology12081054

**Published:** 2023-07-27

**Authors:** Fabio Petrozzi, Sery Gonedele Bi, Gabriel Hoinsoudé Segniagbeto, Nic Pacini, Julia E. Fa, Luca Luiselli

**Affiliations:** 1 Ecolobby, via Edoardo Jenner 70, I-00151 Rome, Italy; fapetrozzi@gmail.com; 2 Laboratoire de Biotechnologie, Agriculture et Valorisation des Ressources Biologiques, UFR Biosciences, Université Félix Houphouet Boigny d’Abidjan-Cocody, Abidjan 22 BP 582, Côte d’Ivoire; sery.gonedele@univ-fhb.edu.ci; 3 Laboratory of Ecology and Ecotoxicology, Faculty of Sciences, University of Lomé, Lomé 01BP1515, Togo; gsegniagbeto@gmail.com; 4 Department of Environmental Engineering, University of Calabria, I-87036 Arcavacata di Rende, Italy; nicopacini@gmail.com; 5 School of Geography, Geology and the Environment, University of Leicester, Leicester LE1 7RH, UK; 6 Department of Natural Sciences, School of Science and the Environment, Manchester Metropolitan University, Manchester M1 5QA, UK; j.fa@mmu.ac.uk; 7 Center for International Forestry Research (CIFOR), Jalan CIFOR, Situ Gede, Sindang Barang, Bogor 16115, Indonesia; 8 Institute for Development, Ecology, Conservation and Cooperation, Via G. Tomasi di Lampedusa 33, I-00144 Rome, Italy; 9 Department of Animal and Environmental Biology, Rivers State University of Science and Technology, Port Harcourt PMB 5080, Nigeria

**Keywords:** freshwater turtles, *Pelusios*, coesxistence, tropical Africa

## Abstract

**Simple Summary:**

Organisms that are similar in size, morphological characteristics, and adaptations, including vertebrates, often coexist by partitioning the available resources (food, space, and time). So, studies of the dynamics of these cases of coexistence are scientifically interesting. Here, we study a coexistence case between two species of freshwater turtles inhabiting the forest waterbodies of West Africa, focusing on the dietary habits of the two species. We found that both turtle species are omnivorous generalists, eating both vegetal and animal matter abundantly. However, there were clear interspecific differences, with the larger of the two species (*P. cupulatta*) eating more vertebrates (mainly fish but occasionally other vertebrates), whereas *P. castaneus* consumed more invertebrates. We also showed that interspecific competition for food does not occur between these two species; instead, previous studies demonstrated that a clear partitioning of the habitat niche occurs.

**Abstract:**

Organisms that are similar in size, morphological characteristics, and adaptations, including vertebrates, often coexist by partitioning the available resources (food, space, and time). So, studies of the dynamics of these cases of coexistence are scientifically interesting. Here, we study a coexistence case of two species of freshwater turtles inhabiting the forest waterbodies of West Africa, focusing on the dietary habits of the two species. We found that both turtle species are omnivorous generalists, eating both vegetal and animal matter abundantly. However, there were clear interspecific differences, with the larger of the two species (*P. cupulatta*) eating more vertebrates (mainly fish but occasionally other vertebrates), whereas *P. castaneus* consumed more invertebrates. These patterns appeared consistently within the species and across sites, highlighting that the same patterns were likely in other conspecific populations from the Upper Guinean forest streams (Côte d’Ivoire and Liberia). Our study also showed that interspecific competition for food does not occur between these two species; instead, previous studies uncovered that a clear partitioning of the habitat niche occurs. We conclude that the food resource is likely unlimited in the study areas, as it is not the case in more arid environments (since food shortages may occur during the dry season). We anticipate that, within the Pelomedusidae communities throughout Africa, intense competition for food probably occurs in the Sahel and Sudanian vegetation zones, particularly during the dry months, but is unlikely within the Guinea and wet savannah region and even less likely in the Guineo-Congolian rainforest region.

## 1. Introduction

Resource partitioning, as described by Chesson [1] and Luiselli [2], provides an explanation for the coexistence of closely related species within the same ecological community without the imminent risk of one species driving the other to extinction through competition. This phenomenon has been supported by studies such as those conducted by Kiel and Peckmann [3] and Nicholson and Clements [4].

Resource partitioning may change seasonally and interannually [5,6]. Moreover, the invasion of a competitor species can push the more specialized one to extinction [7]. Endemic species, which are normally narrowly distributed, may be affected negatively by the invasion of more widespread and generalist species challenging their niche [7,8].

One key aspect of studies of resource partitioning is how the different resources are used by coexisting species. Rather than competing directly for the same resources, these species have evolved to exploit distinct resources, thereby reducing competition. Additionally, resource partitioning can also occur through the utilization of the same resource but in different spatial or temporal niches. This strategy, known as niche partitioning, allows species to coexist by utilizing the shared resource in different ways or at different times [9,10].

Resource partitioning plays a crucial role in both inter- and intra-specific competition dynamics. Inter-specific competition refers to competition between different species, while intra-specific competition involves competition among individuals of the same species. By effectively partitioning resources, species can avoid direct competition and reduce the likelihood of one species outcompeting or displacing another. This allows for the maintenance of species diversity within the community, promoting overall ecological stability and balance [1,9,10].

Although several theories have been used to explain biodiversity patterns, there is no doubt that the reduction of interspecific relative to intraspecific competition can promote the coexistence of species via resource partitioning mechanisms [9,10,11]. Hence, the study of patterns of resource partitioning among species can be used to predict the change in the functioning of ecosystems as a result of the decline in a species [9,10,11].

Freshwater turtles are an ideal group to study resource partitioning in animal communities because they are easily studied and typically form relatively species-poor assemblages, so it is easier to precisely control for the various competitive effects than in species-rich assemblages of species (where, however, other types of analyses are facilitated). In a meta-analysis of freshwater turtle community studies worldwide, the micro-habitat resource was shown to be the most frequently partitioned dimension (in nearly 80% of the study cases), followed by the food resource dimension (nearly 70%) [10]. Competition intensity may negatively affect native turtle species when their niche is invaded by potential competitors, as shown by the impact of the introduced pond slider (*Trachemys scripta*) on the native European pond turtle (*Emys orbicularis*) in Western Europe [10,12].

There are two genera (*Pelomedusa* and *Pelusios*) within the Pelomedusidae family in Sub-Saharan Africa. These turtles are found in a variety of environments but are more common in savannah and forest habitats [10]. Some of these species are widespread generalist species (for instance, *Pelusios castaneus*), whilst others are ecological specialists inhabiting a narrow geographic area (for instance, *Pelusios cupulatta*). In West Africa, some studies have suggested that amongst sympatric Pelomedusidae, “stronger” competitors could be driving “weaker” congenerics into specific habitat patches [13,14,15]. When competition is relaxed (i.e., when the “stronger” competitor does not occur in each area), the “weaker” competitor tends to expand its niche by also colonizing habitats that are not available in sympatry with the “stronger” competitor [13,14]. Moreover, since Pelomedusidae species appear to be carnivorous, feeding on mollusks, crustaceans, aquatic insects, fishes, and amphibians [16,17], their habitat use is heavily conditioned by the availability of these foods; turtle coexistence patterns depend on food abundance in the various habitats [15].

Knowledge of the coexistence dynamics of African Pelomedusidae can shed light on competition and niche partitioning patterns in tropical turtles. In addition, such synecological studies may be important in promoting the conservation of areas where endemic species coexist with widespread or invasive species. West Africa’s Upper Guinean forest zone (Liberia and Côte d’Ivoire) is a biodiversity hotspot where the endemic *Pelusios cupulatta* is found [18,19,20]. This species shares its habitat with two other savannah species (*Pelusios castaneus* and *Pelomedusa variabilis*), which possibly compete with the endemic turtle. These two species (also including other “species” of the *Pelomedusa subrufa* complex) are expanding their range into formerly wooded areas along deforested corridors that have penetrated the forested coastal regions of the Gulf of Guinea, in Côte d’Ivoire (Gonedele Bi et al., unpublished data), and in other West African countries [21]. In some forest creeks in southern Nigeria, *Pelomedusa* and *Pelusios castaneus* have become the dominant species, whereas *Pelusios niger*, a forest specialist, is becoming increasingly rarer, arguably because river banks have been deforested in the last 40 years due to the expansion of the oil and gas industry [21]. The effects of these ecological invasions resulting from massive habitat alterations have not been studied in detail in African turtles. It is possible that the invasion by savannah species will cause a decline in population and worsening of the conservation status of endemic turtles such as *Pelusios cupulatta* [22].

Our a priori hypothesis was that the endemic *Pelusios cupulatta* populations might be affected and therefore decline because of the invasion and apparent increase in numbers by the savannah-dwelling *Pelusios castaneus*. We hypothesized that the negative effect of the savannah species on the forest-specialist endemic turtle might be due to interspecific competition for food and microhabitat, given that the current food supply and pristine microhabitats are likely to be significantly depleted because of human activities for development purposes, together with overfishing with modern techniques. In addition, the use of *P. cupulatta* as food in many local human populations, more often than the other species (Gonedele Bi et al., unpublished data), would increase the relative dominance of the savannah species.

Our objective in this paper was to assess the food composition and the trophic niche resource use of the two study species in a suite of sites where they occur in sympatry as well as in allopatry. We carried out field research during both dry and wet seasons and in different bank and aquatic vegetation types to evaluate if these turtles compete for food. To reach our objectives, we included three types of sites: (a) sites inhabited by only *P. castaneus*, (b) sites inhabited by only *P. cupulatta,* and (c) sites inhabited by sympatric populations of the two species.

## 2. Materials and Methods

### 2.1. Study Species

We focus on two species, *Pelusios castaneus* and *Pelusios cupulatta*. Another species, *Pelomedusa variabilis* (or *subrufa* depending on the authorities), occurs parapatrically with the two above-mentioned species, so it was excluded from our analyses. The two *Pelusios* species are linked to permanent water bodies, whereas *Pelomedusa variabilis* to ephemeral/seasonal ponds [20,21,22]. The two *Pelusios* species, though mostly aquatic, also depend on aquatic and terrestrial habitats for different parts of their life cycles, e.g., predator avoidance, feeding, courtship and mating, basking, and nesting activities. All have a mainly carnivorous, generalist diet but differ considerably in body size (*P. cupulatta* being the larger), which affects prey size selection [22]. *Pelusios cupulatta’s* carapace may easily exceed 30 cm lengthwise, whereas *P. castaneus’s* carapace is usually less than 22 cm long. *Pelusios castaneus* is a widespread species across west-central African savannahs and open forests from southern Senegal, Gambia, Guinea, Guinea-Bissau, Liberia, Sierra Leone, Côte d’Ivoire, Ghana, Togo, Benin, Southern Burkina Faso, southern Niger, Nigeria, Cameroon, south-western Chad, and northern and western Central African Republic [23]. By contrast, *P. cupulatta* is endemic to the Upper Guinean forest region in West Africa, found from Liberia to western Ghana, occurring only within the coastal forest zone [22]. *Pelusios cupulatta*, considered until recently conspecific with *P. niger*, is now described as a distinct species [18].

### 2.2. Study Areas

**Figure 1:**
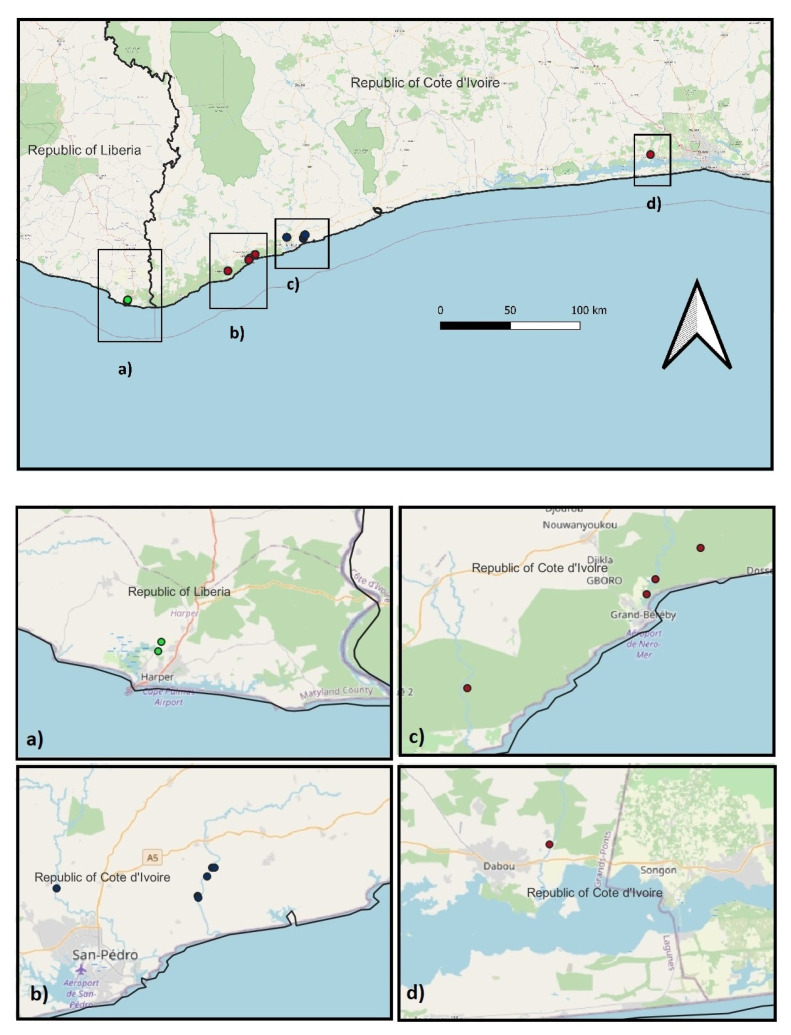
Map of southern Côte d’Ivoire and south-eastern Liberia showing the localities where the capture of turtles was carried out. Green areas indicates forest patches. Red dots: sympatric populations; dark blue dots: *Pelusios castaneus*; light blue dots: *Pelusios cupulatta*. In this plate, (**a**) sites with the presence of only *P. cupulatta* in the Harper region of Liberia; (**b**) sites with the presence of only *P. castaneus* in the San Pedro area of Côte d’Ivoire; (**c**) sites with the presence of both species in the Sassandra-San Pedro area of Côte d’Ivoire; (**d**) sites with the presence of both species in the Abidjan area of Côte d’Ivoire.

Our study areas were in the southern part of Côte d’Ivoire (in particular in the Sassandra and San Pedro areas) and in the Harper area of south-eastern Liberia (Figure 1), where we have already surveyed turtle populations in recent years and hence established where one or two species are found [14]. These localities are similar in climate and habitat characteristics, with the highest rainfall in the country (an average of >1800 mm) [14], and are ideal for our study given our existing links with the local human communities, whose economy consists principally of agriculture, fisheries, and mineral extraction. Other activities threaten the environment of this area. These include the clearing of coastal vegetation (particularly mangroves), the removal of sand and dam building on rivers for agriculture or energy production, the expansion of makeshift buildings to accommodate the increasing human population, as well as an emerging tourism business.

Five biome types are typical within the study area: (1) coastal forest; (2) swamp forest; (3) mangrove; (4) prelagunar savannas, and (5) coastal savannas. The coastal forest is an evergreen forest formation. This dense humid forest and its streams/wetlands (typical habitats for *P. cupulatta*) accounted for >13 million hectares along the coast and its immediate hinterland in 1955 but had declined to <900,000 hectares by 2000. This amounts to nearly 94% of forest lost in 45 years. Most forest has been replaced by plantations, grasslands, or fallows (typical habitats for *P. castaneus* and *Pelomedusa* sp.) [14].

Three types of study areas were selected to investigate the trophic niche of the two species: site-type A and B, where alternatively each of the two species was found (respectively, only *P. castaneus* and *P. cupulatta*), and site-type C, where the two species were sympatric with an apparently similar abundance (Figure 1). It cannot be categorically excluded that the absence of one of the two species at the sites of “absence” is real or apparent—that is, that the species is present at the site but with extremely low density (as it has never been observed throughout our pilot studies, i.e., 7-day surveys with trapping at each site, from 2014 to 2022). Consequently, their ecological interactions/effects on the more abundant populations of the other species are probably of little consequence.

All study areas are within the same geographical province of Côte d’Ivoire and Liberia and are similar in terms of bioclimate but differ in terms of the type of bank vegetation (less forested in the *P. castaneus*-only site and more forested in the other three areas).

### 2.3. Protocol

At each site, we organized a system of trapping using turtle traps in all available microhabitats. For each study site, the traps were set for one month during the dry and wet seasons (July and December 2022). Each trap, constructed using fine mesh, was approximately 120 to 180 cm in length and had a hoop diameter of about 91 cm. In most cases, we placed the same number of traps (*n* = 30) baited with fish at each site and each day. The fine mesh size prevented the legs of the turtles from becoming entangled. The top of the traps remained above water to allow entrapped turtles to obtain air. All traps were checked daily. No captured turtles died during our study.

Each captured individual was identified to species and sexed, measured (midline carapace and plastron length, as well as weight), and marked by notching a scute. We analyzed the food ingested by the animals using fecal analysis [24], keeping the various individuals in a container until defecation occurred. Food item remains were identified with the use of a dissecting microscope and analyzed to the lowest taxonomical level possible. Additional in situ direct observations of feeding turtles were added to the data and used for analyses. For dietary analyses, we considered the number of individuals containing a given food type and not the percentage contribution (in terms of the number of items or biomass) of each food item to the diet of each individual.

### 2.4. Statistical Analyses

For our analyses, we defined as (i) CAS1 and CAS2, the *P. castaneus* populations sympatric with the potential competitor in San Pedro-Sassandra and Abidjan areas respectively; (ii) CUP1 and CUP2, *P. cupulatta* populations sympatric with the potential competitor in San Pedro-Sassandra and Abidjan areas respectively; (iii) CAS_ALL and CUP_ALL, allopatric populations of the two species (see Online Supplementary Table S1 for the raw data). Food niche overlap between species in the various conditions of sympatry/allopatry was calculated using Pianka’s [25] symmetric equation, with values ranging from 0 (no overlap) to 1 (total overlap). Pianka’s values were calculated using Past 4.0 statistical software, the randomization algorithms RA2 and RA3, with 30,000 random Monte Carlo simulations (model’s resource state = equiprobable) [26,27,28]. Frequency differences in terms of dietary items between species were assessed by contingency-table chi-square tests. Differences in food niche overlap between the two species when sympatric vs. allopatric were analyzed using the Mann–Whitney U-test.

In order to evaluate whether the dietary composition of the two turtle species was adequately described by the collected data, we used a saturation curve with the number of examined individuals (*X* axis) against the cumulative number of different food items recorded (*Y* axis). Using this approach, the dietary composition of turtles at each site is adequately described by the collected data if the saturation curve reaches a plateau phase.

All statistical analyses were performed with PAST, Statistica, SPSS, and EcoSim software. Alpha was set at 5%, and all tests were two-tailed. In the analyses, we used the number of individuals containing a given food type and not the percent of a given food type in the gut contents of each individual turtle.

## 3. Results

Summarized data are given in the Online Supplementary Table S1. The number of examined individuals per species and per site type ranged from 24 to 41, with a total of 113 *P. castaneus* and 87 *P. cupulatta* analyzed.

**Figure 2:**
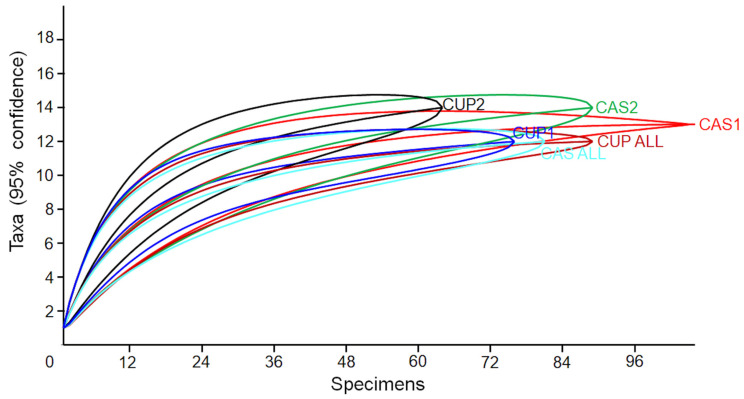
Saturation curves for the various populations surveyed during the present study. The 95% confidence intervals, obtained after 9999 bootstraps, are also presented. Symbols: CAS1 and CAS2, *P. castaneus* populations sympatric with the potential competitor; CUP1 and CUP2, *P. cupulatta* populations sympatric with the potential competitor; CAS_ALL and CUP_ALL, allopatric populations of the two species.

Although the number of individuals per site and per species was relatively low, analysis of saturation curves showed that the food niche breadth of all the populations, in both sympatric and allopatric conditions, reached a plateau phase of eaten foods in all cases (Figure 2).

In terms of the percent of individuals containing a given prey type, we observed the following patterns: in Sassandra-San Pedro sites (see Figure 1c))where the two *Pelusios* species occurred sympatrically, there were only three food categories found in the various individuals which differed significantly between species (*p* < 0.05 at χ^2^ test): aquatic plants, insects, and fish. The first two food categories occurred significantly more often in *P. castaneus,* whereas the latter food type was found significantly more often in *P. cupulatta* (Online Supplementary Figure S1). Pianka’s value of trophic niche overlap between the two species was 0.601.

**Figure 3:**
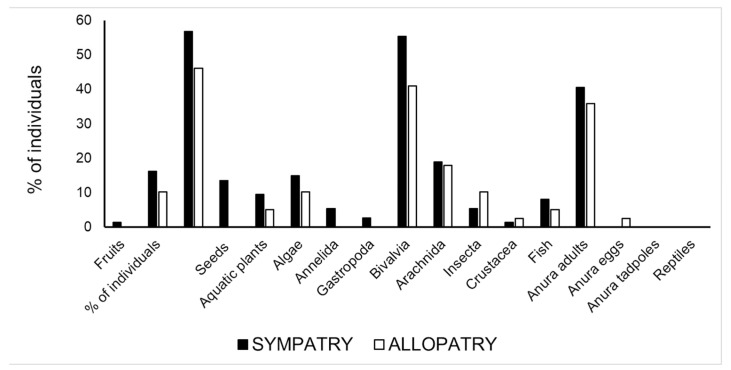
Percentage of turtles containing a given food type in sympatry vs. allopatric conditions in *P. castaneus*.

**Figure 4:**
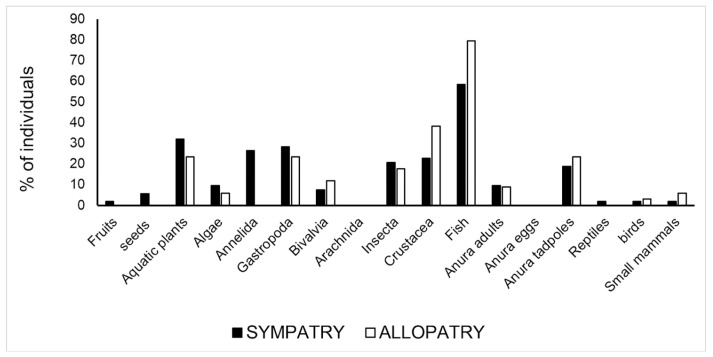
Percentage of turtles containing a given food type in sympatry vs. allopatric conditions in *P. cupulatta*.

In Abidjan area, where the two *Pelusios* species occurred sympatrically, there were four food categories that were found in various individuals that significantly differed between species (*p* < 0.05 at χ^2^ test): aquatic plants, insects, fish, and anuran tadpoles (Figure S2). The first two categories and the anuran tadpoles occurred significantly more often in *P. castaneus,* whereas fish was found significantly more often in *P. cupulatta* (Figure S2). At these sympatry sites, the two species consistently partitioned three food type categories: aquatic plants, insects, and fish (compare the patterns in Figure 3 and Figure 4). Pianka’s value of trophic niche overlap between the two species was 0.638.

In sites where the two *Pelusios* species occurred in allopatry (Figure S3), the same patterns highlighted above were observed: aquatic plants, insects, and anuran tadpoles were the main food types for *P. castaneus* and fish, followed by crustaceans and aquatic plants, which were the main food types of *P. cupulatta*. Pianka’s value of trophic niche overlap between the two species, when comparing their diet breadth in allopatric conditions (i.e., CAS_ALL vs. CUP_ALL), was 0.579. Concerning *P. castaneus*, when we compared CAS1 with CAS2, Pianka’s value of niche overlap was 0.961, CAS1 with CAS_ALL was 0.962, and CAS2 with CAS_ALL was 0.980.

For *P. cupulatta*, when we compared CUP1 with CUP2, Pianka’s value of niche overlap was 0.916, CUP1 with CUP_ALL was 0.915, and CUP2 with CUP_ALL was 0.878. These Pianka’s niche overlap estimates showed that: (1) the food habits of the various turtle populations were significantly more similar within species than between species (*p* < 0.05 at Mann–Whitney U-test), but the food niche overlap did not vary significantly (*p* > 0.312 at Mann–Whitney U-test) between the two species when the sympatric vs. the allopatric populations of both *P. castaneus* and *P. cupulatta* were compared. There was no apparent effect of sympatry on the niche overlap of the species, showing no sign of interspecific food competition.

As a confirmation of the above-mentioned pattern, Lawlor’s RA3 algorithm indicated no evidence of non-random structure (interspecific competition) for food between the two study species (observed mean index = 0.740) using both RA3 (mean of simulated indices = 0.39818; variance of simulated indices = 0.00146; random seed = −32,331,781; *p*_(observed <= expected)_ = 0.996; *p*_(observed >= expected)_ = 0.004) and RA2 (random seed = 1,490,256,373; *p*_(observed <= expected)_ = 0.99100; *p*_(observed >= expected)_ = 0.00900).

## 4. Discussion

We confirm that both turtle species are omnivorous generalists, eating both vegetal and animal matter abundantly. However, there were clear interspecific differences, with the larger of the two species (*P. cupulatta*) eating more vertebrates (mainly fish but occasionally other vertebrates), whereas *P. castaneus* consumed more invertebrates. These patterns appeared consistently within the species and across sites, highlighting that the same patterns were likely in other conspecific populations from the Upper Guinean forest streams (Côte d’Ivoire and Liberia). These differences seem to be correlated with the respective body size, that is far larger in *P. cupulatta* so that it can feed on remarkably larger prey items than *P. castaneus*. Since it is well known that freshwater turtles also feed on carrion, the fact that only the largest species has fed on vertebrates and prey of a certain size suggests that *P. cupulatta* also actively preys on small vertebrates, both terrestrial and aquatic, and not simply that it feeds on them once they die. From a qualitative point of view, the generalist predominantly carnivorous diet of the two *Pelusios* species is not surprising, as it has been demonstrated in earlier studies with several species of the same genus in various African ecosystems [17].

The most interesting evidence of our study is that interspecific competition for food does not likely occur between these two species; instead, previous studies revealed a clear partitioning of the habitat niche between these two species [14]. We hypothesize, therefore, that the food resource is not limited in the study areas, as is probably the case in more arid environments (where food shortages may occur during the dry season). An interesting comparison can be created between our study system from the rainforest region of West Africa and the study system of other pelomedusid turtles from the arid and semiarid regions of Africa. We suggest that, within the pelomedusid communities throughout Africa, intense competition for food probably occurs in the Sahel and Sudanian vegetation zones, particularly during the dry months, but this is unlikely within Guinea and the wet savannah region and even less likely in the Guineo-Congolian rainforest region (our study). In fact, at a continental scale, the various Pelomedusidae species co-occurred following assembly rules that are compatible with interspecific competition at the habitat scale, with body size filtering also being a mechanism that facilitates their coexistence under sympatric conditions [29]. Indeed, in our study, *P. cupulatta* was significantly larger than *P. castaneus* [29].

An alternative hypothesis is that these two species may co-occur in the study areas because they simply already (before contact) were different enough in diet to allow coexistence. According to this “historical” hypothesis, the fact that their diets change little in allopatry argues against partitioning as a process that arose out of the need for sympatry.

In addition, because our sampling locations are sometimes far apart (>300 km in a few cases), we could not definitely disentangle the effects of habitat vs. the effects of competition on diet composition. The similarity in habitat characteristics, even among the most distant localities, suggests, however, that the distance may have been a minor factor for the given data.

Another potential issue with the interpretation of the observed patterns derives from the methodology used and its limitations [24]. Indeed, the difference in diet between species may also be because fecal analysis only provides a snapshot of an animal’s last meal. Given that both species appear to have a very broad diet, the differences between species may just reflect fluctuations in local abundance in some prey rather than actual differences in prey preference. Isotopic niche analyses may be useful to supplement our fecal pellet analyses in order to better describe the diet characteristics of these turtle species.

In terms of the conservation of turtles in West Africa, we suggest that the analysis of food niches, at least for the two species in our study, does not infer interspecific competition for food and may not be affected by the presence of the potential competitor in terms of trophic niche/limitation. Thus, it is unlikely that the generalist *P. castaneus* may influence the presence of the endemic *P. cupulatta* via any superior competitive ability. Since habitat alteration and human consumption of turtles are potential explanations for the decline in *P. cupulatta* in the presence of colonizing *P. castaneus*, it would be important to pay more attention to investigating these potential threats other than the aforementioned hypotheses.

## Data Availability

All data pertinent to this study are presented herein.

## Supplementary Materials

The following supporting information can be downloaded at: https://www.mdpi.com/article/10.3390/biology12081054/s1. Table S1. Synopsis of the food types eaten by the various turtle populations. Figure S1. Percentage of turtles containing a given food type in a sympatry area (Sassandra-San Pedro, Côte d’Ivoire) of *P. castaneus* and *P.cupulatta*. Figure S2. Percentage of turtles containing a given food type in a sympatry site (Abidjan area, Côte d’Ivoire) of *P. castaneus* and *P. cupulatta*. Figure S3. Percentage of turtles containing a given food type in allopatric condition.

## References

[1] Chesson P. (2000). Mechanisms of maintenance of species diversity. Annu. Rev. Ecol. Evol. Syst..

[2] Luiselli L. (2006). Food niche overlap between sympatric potential competitors increases with habitat alteration at different trophic levels in rain-forest reptiles (omnivorous tortoises and carnivorous vipers). J. Trop. Ecol..

[3] Kiel S., Peckmann J. (2019). Resource partitioning among brachiopods and bivalves at ancient hydrocarbon seeps: A hypothesis. PLoS ONE.

[4] Nicholson G.M., Clements K.D. (2020). Resolving resource partitioning in parrotfishes (Scarini) using microhistology of feeding substrata. Coral Reefs.

[5] Luiselli L. (2006). Resource partitioning and interspecific competition in snakes: The search for general geographical and guild patterns. Oikos.

[6] Luiselli L. (2006). Interspecific relationships between two species of sympatric Afrotropical water snake in relation to a seasonally fluctuating food resource. J. Trop. Ecol..

[7] Wauters L., Tosi G., Gurnell J. (2005). A review of the competitive effects of alien grey squirrels on behaviour, activity and habitat use of red squirrels in mixed, deciduous woodland in Italy. Hystrix.

[8] Luiselli L. (2001). The ghost of a recent invasion in the reduced feeding rates of spitting cobras during the dry season in a rainforest region of tropical Africa?. Acta Oecol..

[9] Schoener T.W. (1974). Resource Partitioning in Ecological Communities: Research on how similar species divide resources helps reveal the natural regulation of species diversity. Science.

[10] Luiselli L. (2008). Resource partitioning in freshwater turtle communities: A null model meta-analysis of available data. Acta Oecol..

[11] Begon M., Towsend C.R. (2021). Ecology.

[12] Romero D., Báez J.C., Ferri-Yáñez F., Bellido J.J., Real R. (2014). Modelling Favourability for Invasive Species Encroachment to Identify Areas of Native Species Vulnerability. Sci. World J..

[13] Luiselli L., Akani G.C., Ajong S.N., George A., Di Vittorio M., Eniang E.A., Dendi D., Hema E.M., Petrozzi F., Fa J.E. (2020). Predicting the structure of turtle assemblages along a megatransect in West Africa. Biol. J. Linn. Soc..

[14] Petrozzi F., Ajong S.N., Pacini N., Dendi D., Gonedele Bi S., Fa J.E., Luiselli L. (2021). Spatial Niche Expansion at Multiple Habitat Scales of a Tropical Freshwater Turtle in the Absence of a Potential Competitor. Diversity.

[15] Gbewaa S.B., Oppong S.K., Horne B.D., Tehoda P., Petrozzi F., Dendi D., Akani G.C., Di Vittorio M., Ajong S.N., Pacini N. (2021). Community Characteristics of Sympatric Freshwater Turtles from Savannah Waterbodies in Ghana. Wetlands.

[16] Lemell P., Beisser C.J., Weisgram J. (2000). Morphology and function of the feeding apparatus of *Pelusios castaneus* (Chelonia; Pleurodira). J. Morphol..

[17] Luiselli L., Demaya G.S., Benansio J.S., Petrozzi F., Akani G.C., Eniang E.A., Ajong S.N., Di Vittorio M., Amadi N., Dendi D. (2021). A Comparative Analysis of a Genus of Freshwater Turtles Across Africa. Diversity.

[18] Rhodin A.G.J., Iverson J.B., van Dijk P.P., Saumure R.A., Buhlmann K.A., Pritchard P.C.H., Mittermeier R.A., Turtle Taxonomy Working Group (2017). Turtles of the World: Annotated Checklist and Atlas of Taxonomy, Synonymy, Distribution, and Conservation Status. Conservation Biology of Freshwater Turtles and Tortoises: A Compilation Project of the IUCN/SSC Tortoise and Freshwater Turtle Specialist Group.

[19] Luiselli L., Diagne T., Mcgovern P. (2021). Prioritizing the next decade of freshwater turtle and tortoise conservation in West Africa. J. Nat. Conserv..

[20] McGovern P., Luiselli L. (2023). Knowledge gaps and conservation priorities for west African chelonians. Amphibia-Reptilia.

[21] Luiselli L., Angelici F.M., Politano E. (2000). Ecological correlates of the distribution of terrestrial and freshwater chelonians in the Niger Delta, Nigeria: A biodiversity assessment with conservation implications. Rev. Ecol. (Terre et Vie).

[22] Branch B. (2008). Tortoises, Terrapins and Turtles of Africa.

[23] Bour R., Luiselli L., Petrozzi F., Segniagbeto G.H., Chirio L., Rhodin A.G.J., Pritchard P.C.H., van Dijk P.P., Saumure R.A., Buhlmann K.A., Iverson J.B., Mittermeier R.A. (2016). *Pelusios castaneus* (Schweigger 1812)—West African Mud Turtle, Swamp Terrapin. Conservation Biology of Freshwater Turtles and Tortoises: A Compilation Project of the IUCN/SSC Tortoise and Freshwater Turtle Specialist Group.

[24] Luiselli L., Amori G., Dodd C.K. (2016). Diet. Reptile Ecology and Conservation: A Handbook of Techniques.

[25] Pianka E.R. (2017). Ecology and Natural History of Desert Lizards: Analyses of the Ecological Niche and Community Structure.

[26] Gotelli N.J., Graves G.R. (1996). Niche Overlap. Null Models in Ecology.

[27] Solida L., Celant A., Luiselli L., Grasso D.A., Mori A., Fanfani A. (2011). Competition for foraging resources and coexistence of two syntopic species of *Messor* harvester ants in Mediterranean grassland. Ecol. Entomol..

[28] Djagoun C.A.M.S., Codron D., Sealy J., Mensah G.A., Sinsin B. (2016). Isotopic niche structure of a mammalian herbivore assemblage from a West African savanna: Body mass and seasonality effect. Mamm. Biol..

[29] Luiselli L., Di Vittorio M., Rhodin A.G., Iverson J.B. (2021). Variation of community assembly rules of a whole turtle family (Pelomedusidae) from continental to local scales in Africa. Ecol. Res..

