# Supplementary material for: Trophic Resource Use by Sympatric vs. Allopatric Pelomedusid Turtles in West African Forest Waterbodies
Source: Biology (Basel). 2023 Jul 27;12(8):1054. doi: 10.3390/biology12081054 (PMC10451615; doi:10.3390/biology12081054)

biology

MDPI

ONLINE SUPPLEMENTARY MATERIALS

Table S1. Synopsis of the food types eaten by the various turtle populations. Note that, in this table, the number of individuals containing a given food type, and not the total number of food items, is considered. Abbreviations: CAS1 and CAS2, *P. castaneus* populations sympatric with the potential competitor; CUP1 and CUP2, *P. cupulatta* populations sympatric with the potential competitor; CAS_ALL and CUP_ALL, allopatric populations of the two species, N = total number of individuals per site

	CAS1	*CUP1*	*CAS2*	*CUP2*	*CAS_ALL*	*CUP_ALL*
N	41	29	33	24	39	34
Fruits	0	0	1	1	0	0
Seeds	7	0	5	3	4	0
Aquatic plants	23	9	19	8	18	8
Algae	5	5	5	0	0	2
Annelida	4	8	3	6	2	0
Gastropoda	8	9	3	6	4	8
Bivalvia	3	1	1	3	0	4
Arachnida	2	0	0	0	0	0
Insecta	24	4	17	7	16	6
Crustacea	6	7	8	5	7	13
Fish	2	18	2	13	4	27
Anura adults	0	2	1	3	1	3
Anura eggs	2	0	4	0	2	0
Anura tadpoles	13	8	17	2	14	8
Reptiles	0	0	0	1	1	0
Birds	0	0	0	1	0	1
Small mammals	0	1	0	0	0	2
Indeterminate	7	4	3	5	8	7

Figure S1. Percentage of turtles containing a given food type in a sympatry area (Sassandra-San Pedro, Côte d'Ivoire) of *P. castaneus* and *P.cupulatta*.

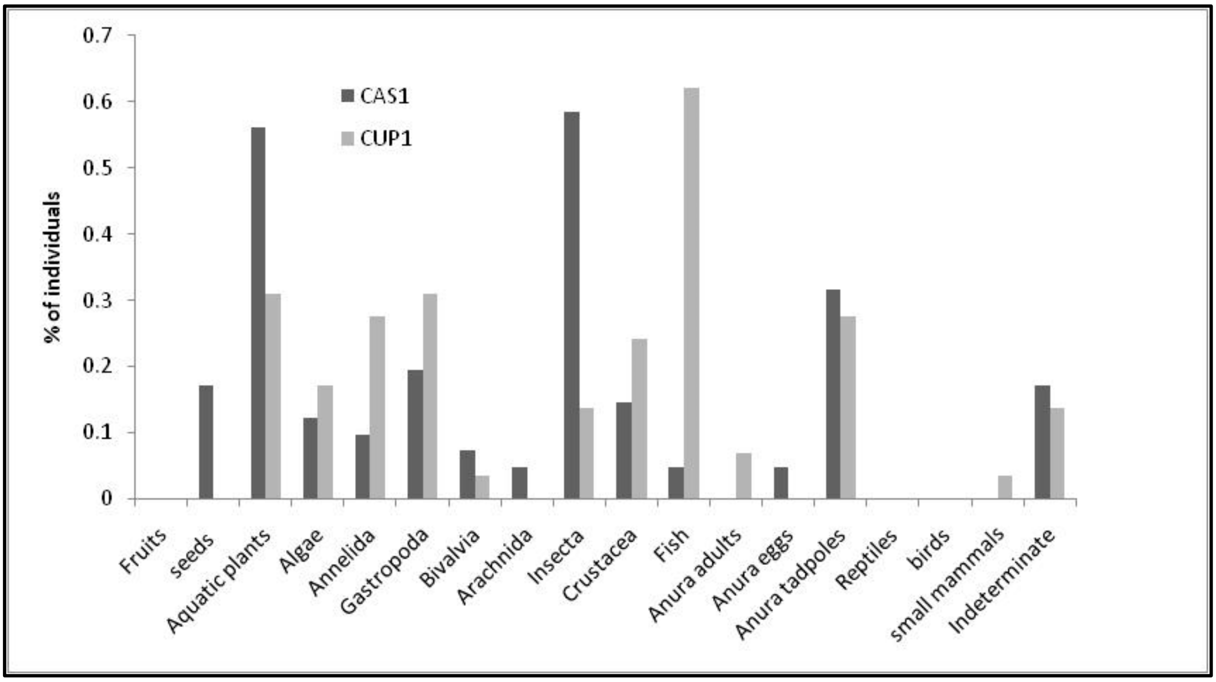

Figure S2. Percentage of turtles containing a given food type in a sympatry site (Abidjan area, Côte d'Ivoire) of *P. castaneus* and *P. cupulatta*.

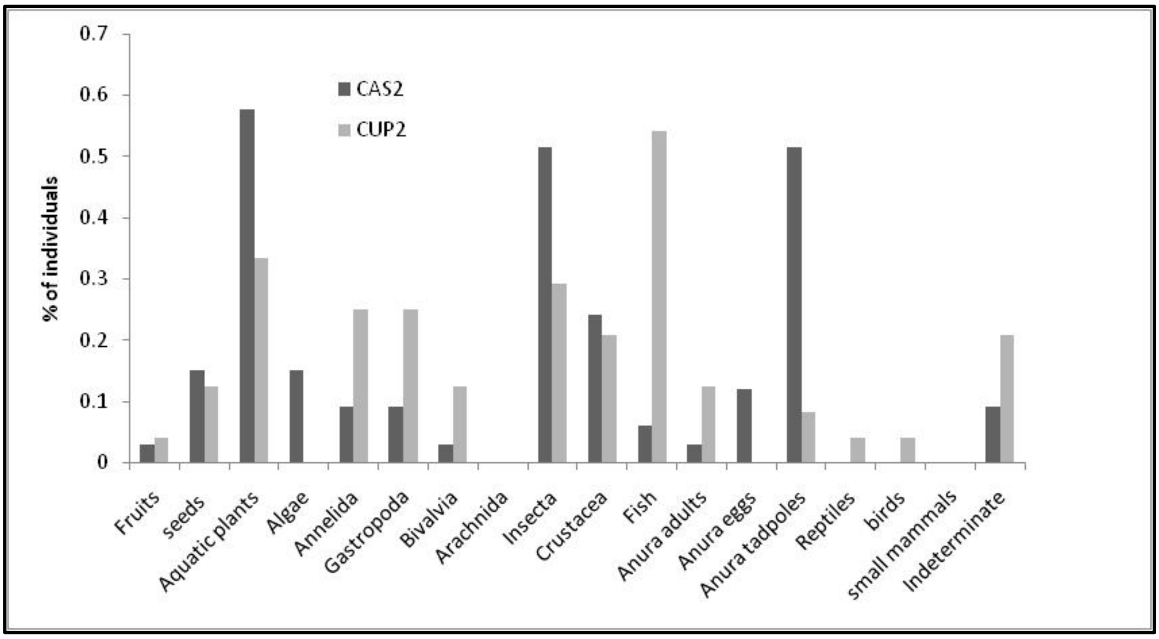

Figure S3. Percentage of turtles containing a given food type in allopatric condition.

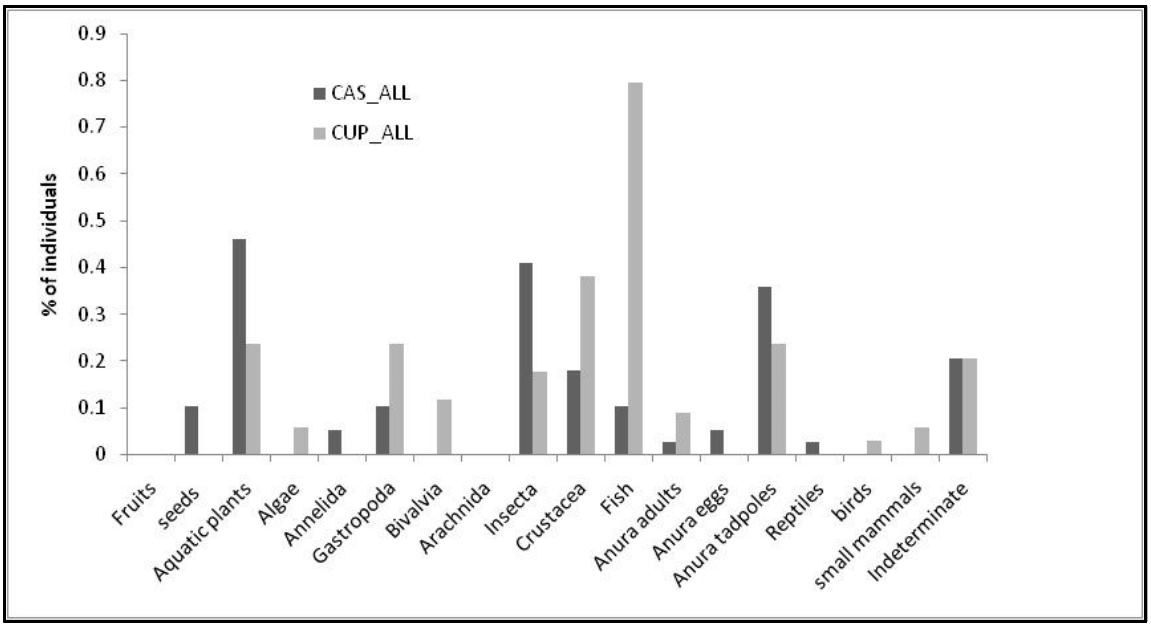

Supplement: Supplementary file 1 [file biology-12-01054-s001.zip › biology-2456173-supplementary.pdf]
